# HIV and SARS-CoV-2 Co-Infection: From Population Study Evidence to In Vitro Studies

**DOI:** 10.3390/life12122089

**Published:** 2022-12-13

**Authors:** Chiara Stefani, Tobia Fantoni, Michele Bissoli, Jordan Thomas, Alessandra Ruggiero

**Affiliations:** 1 Section of Biology and Genetics, Department of Neurosciences, Biomedicine and Movement Sciences, University of Verona, 37134 Verona, Italy; 2 Department of Clinical Infection, Microbiology and Immunology, Institute of Veterinary and Ecological Sciences, University of Liverpool, Liverpool L69 3BX, UK

**Keywords:** HIV-1, SARS-CoV-2, co-infections, anti-retroviral drugs

## Abstract

Human immunodeficiency virus type 1 (HIV-1) and severe acute respiratory syndrome coronavirus 2 (SARS-CoV-2) have caused two major viral outbreaks during the last century. Two major aspects of HIV-1 and SARS-CoV-2 co-infection have been extensively investigated and deserve attention. First, the impact of the co-infection on the progression of disease caused by HIV-1 or SARS-CoV-2. Second, the impact of the HIV-1 anti-retroviral treatment on SARS-CoV-2 infection. In this review, we aim to summarize and discuss the works produced since the beginning of the SARS-CoV-2 pandemic ranging from clinical studies to in vitro experiments in the context of co-infection and drug development.

## 1. Introduction

Human immunodeficiency virus type 1 (HIV-1) and severe acute respiratory syndrome coronavirus 2 (SARS-CoV-2) are two of the most impactful viral outbreaks of the past century. Both outbreaks have caused substantial global mortality as well as significant and far reaching social and economic consequences.

HIV-1 and SARS-CoV-2 are RNA viruses that induce an excessive inflammatory status with a high risk of mortality, albeit with a different magnitude between the two viruses, for vulnerable individuals, including the elderly, newborns, pregnant women and immunocompromised subjects [1,2]. The pathogenesis of the two viruses is on opposite fronts: HIV-1 targets the cells of the immune system with an initial latent infection that induces the progressive depletion of CD4+ T lymphocytes, thus compromising the coordination of the immune system. This results in acquired immunodeficiency syndrome (AIDS) if the infection is not treated in time. On the other hand, SARS-CoV-2 induces predominantly acute infections of respiratory tract tissues, resulting in the development of pneumonia and acute respiratory distress syndrome (ARDS) in the most severe cases [3]. The main characteristics of the two examined viruses are reported in Table 1.

### 1.1. Human Immunodeficiency Virus-1 (HIV-1)

HIV-1 belongs to the *Retroviridae* family, a group of viruses characterized by their ability to convert their RNA genome into double-stranded DNA through their retro-transcribing polymerase [4]. The primary target cells of HIV-1—CD4+ T lymphocytes—are essential participants in cell-mediated adaptive immune responses.

HIV-1 gp120 protein binds the CD4 receptor and one of the co-receptors (CCR5/CXCR4) on the target cell, facilitating virus and cell membrane fusion and subsequent entry into the target cell, starting the process of reverse transcription and integration into the host DNA [4]. HIV-1 infection begins with an acute phase, initiated within the first week following infection, during which the patient presents flu-like symptoms and high viremia, with substantial depletion of memory T cells in gut-associated T lymphoid tissue (GALT) and lymph nodes. After this acute phase, the viremia reaches a set point and the chronic infection is initiated, resulting in the progressive loss of T helper lymphocytes [5]. The patient reaches AIDS when the CD4+ T cells count is less than 200 cell/mm^3^ in the blood. AIDS is characterized by recurring opportunistic infections that can lead to the patient’s death [6]. Up to now, the only treatment available to limit HIV infection is the use of anti-retroviral drugs, molecules that can inhibit different HIV life-cycle steps, entry inhibitors (prevent membrane fusion and HIV entry, such as CCR5 inhibitors), the reverse-transcriptase inhibitors (block the reverse transcriptase process, such as nucleotide/nucleoside or non-nucleotide/nucleoside inhibitors), integrase inhibitors and protease inhibitors (prevent HIV genome integration and HIV protein cleavage) [7]. Combination anti-retroviral therapy (cART) prevents the emergence of drug resistance within HIV infected patients and is therefore more effective than single-drug-based therapies. Following the advent of cART, the life expectancy of people living with HIV (PLWH) is substantially improved; however, neither a cure to eradicate HIV nor an effective vaccine exist [8,9]. Further, global statistics on HIV-1 in 2021 reported that there were 38.4 million PLWH (1.5 million newly infected), yet only 28.7 million people globally had access to cART [10].

### 1.2. Severe Acute Respiratory Syndrome-Coronavirus-2 (SARS-CoV-2)

SARS-CoV-2 is a novel coronavirus isolated for the first time in the province of Wuhan, Hubei, China at the end of 2019. The virus is highly transmissible and therefore rapidly became pandemic with, until now, 550 million confirmed cases and almost 6.3 million deaths [11]. SARS-CoV-2 is a respiratory virus that predominantly infects the upper respiratory tract, albeit some variants show high affinity for the lower respiratory tract and, as such, a greater propensity to induce pneumonia. The spike protein trimers on its envelope mediates virus entry via interaction with the human angiotensin-converting enzyme 2 (ACE2) which acts as receptor for spike. After binding, virus entry is completed when the virus fusion complex is induced by an enzymatic cleavage performed by the human transmembrane serine protease 2 (TMPRSS) [12]. After the uncoating event, the single stranded positive RNA genome is replicated and translated into protein, after which viral assembly leads to the release of complete virus particles. SARS-CoV-2 infection is associated with a cytokine storm that causes massive inflammation of lung tissue with the development of interstitial pneumonia and the presentation of the coronavirus disease, named COVID-19 [1,3]. Patients with overactivated immune responses suffer from the most severe respiratory conditions, thus requiring artificial ventilation to survive [13]. Due to its RNA genome and the absence of a proofreading activity in the viral RNA-dependent RNA polymerase, the SARS-CoV-2 genome has accumulated substantial diversity since its outbreak into the human population. In particular, mutations in the spike protein coding region potentially confer immune escape and can result in a decreased vaccine-mediated protection, even leading to non-protection [14,15,16]. Owing to the substantial prevalence of SARS-CoV-2 in the general population, co-infection with SARS-CoV-2 amongst PLWH is likely common, though the consequences of this condition are less understood than mono-infection with either virus. In this review, we will provide an overview of the clinical studies to discuss the implications associated with HIV-1 and SARS-CoV-2 co-infection. Furthermore, we will focus on the co-infection event and the clinical outcomes, with a few examples to further understand the pharmaceutical therapy that PLWH must follow and some possible cross action in the SARS-CoV-2 infection.

**Table 1:** Sum up of differences and similarities between HIV-1 and SARS-CoV-2.

Disease Caused by the Virus	HIV-1	SARS-CoV-2
	AIDS	COVID-19
Genome organization and translation mechanism	RNA genome with a cDNA intermediate followed by integration in the host genome [4]	ssRNA positive genome translated in protein immediately after the infection [2]
Target cells	T cells, macrophages, astrocytes, microglia cells [17]	Respiratory tissues, kidneys, small intestines, pancreas, blood vessels [18]
Target receptor/coreceptor	CD4/CCR5 and CXCR4	ACE2/TMPRSS2
Viral persistence	Lifelong infection that can be managed with cART [5]	From 10 to 17 days [19]
Suggested treatment	Combination of anti-retroviral drugs to avoid the adaptation of HIV-1 to the treatment [8]	Antipyretics, corticosteroids, immunomodulatory agents, mAbs, antivirals, based on the disease severity [20]

## 2. Population Studies on HIV-1 and SARS-CoV-2 Co-Infections: A Global Overview

The COVID-19 pandemic has had a profound impact on the healthcare system with regard to the treatment of other morbidities and infections. It has been reported that 60% of infectious disease physicians were working on COVID-19 patients, resulting in about 70% of HIV-1 treatment facilities in Central and Eastern Europe not being operative, reducing the ability to offer appropriate treatment and care to PLWH [21,22]. Therefore, access to anti-retroviral therapies and facilities dedicated to HIV-1 became more difficult for PLWH.

It is still debated whether PLWH have a higher risk of infection or severe complications due to SARS-CoV-2 or if they are protected by cART or by the immune suppression status as a result of HIV-1 infection. Comorbidities derived from HIV-1 infection such as diabetes, hypertension, obesity, cardiovascular, renal and lung diseases could represent risk factors for severe SARS-CoV-2 infection. Conversely, the reduced immune response that characterizes advanced HIV-1 infection could be protective against severe complications of SARS-CoV-2 infection [23]. In a cohort of 30 PLWH hospitalized for SARS-CoV-2 complications in France, 90% of them were virologically suppressed and had comorbidities such as cardiovascular and renal diseases, hypertension and diabetes. The authors concluded that HIV-1 is not an independent risk factor for SARS-CoV-2 infection [24]. Other groups explored SARS-CoV-2 seroprevalence in the HIV-1 population. Noe et al. performed a study on a German ‘hot-spot’ area that included a population of 500 PLWH and concluded that the rate of seroprevalence in PLWH does not seem to exceed previous reports from the general population [25]. In a retrospective study conducted in Germany, a group of 33 PLWH undergoing cART with mainly reverse transcriptase inhibitors who were co-infected with SARS-CoV-2 were evaluated for COVID-19 symptomatology. They found the following most common comorbidities: hypertension (10 out of 33 patients), chronic obstructive pulmonary disease (6 out of 33 patients), diabetes (4 out of 33), cardiovascular disease (3 out of 33) and renal insufficiency (2 out of 33). The most frequent symptoms included: cough, fever and arthralgia. Only 24% of these patients were classified as severe cases and three patients died. The study concluded that there is not an increased risk of mortality and morbidities in cART-treated PLWH with symptomatic COVID-19 infection. However, the authors of these studies underlined some important limitations such as the lack of asymptomatic cases, the absence of transmission and exposure information as well as the limited data on the onset, intensity and duration of symptoms [26]. Another study analyzed 51 PLWH diagnosed with COVID-19 in 2020 in Spain. The rate of COVID-19 infection among PLWH periodically followed up was 1.8%. Moreover, an increased incidence of comorbidities was found in HIV-1- and SARS-CoV-2-positive patients compared with only HIV-1-positive or SARS-CoV-2-positive subjects. Since 25% of patients had severe COVID-19 disease and 12% were admitted to the intensive care unit, the authors concluded that PLWH might have worse outcomes. Moreover, they did not identify an association between cART and COVID-19 severity [27]. A large English study compared data from 207 different centers located in UK. Among the 47,592 analyzed subjects, only 0.26% were HIV-1 positive. Importantly, the authors concluded that mortality rate was increased in PLWH under 60 years old compared with HIV-1 uninfected individuals [28]. Another large study included 77,590 PLWH undergoing cART in Spain. This work suggested that whilst HIV-1-positive men over 70 years old showed a higher risk for COVID-19 diagnosis, the use of non-nucleoside reverse transcription inhibitors (NNRTIs) TDF/FTC appeared to be protective for COVID-19 hospitalization [29].

This latter evidence paves the way for further debate on the use of anti-retrovirals against HIV-1 and their use in COVID-19 therapy [30]. For example, the role of protease inhibitors (PIs) has been evaluated. It has been reported that the treatment using ritonavir/lopinavir in combination with oseltamivir (a drug used in flu treatment) had a positive effect on a 71-year-old woman with a severe SARS-CoV-2 infection in Thailand [30]. However, this drug regimen failed in a trial performed in China on 199 SARS-CoV-2 patients. The subjects were divided into two groups: one taking the standard drug regimen, the other taking the standard drug regimen and PIs (ritonavir/lopinavir). The study concluded that there is no difference in the SARS-CoV-2 disease outcome between the two study groups and that the observed mortality rate was similar [31]. Another study on the effectiveness of ritonavir/lopinavir in 47 patients in China demonstrated that PIs in combination with adjuvant drugs have positive effects on recovery, including decreasing body temperature and on recovering physiological mechanisms [32]. Other drug regimens that have been studied through phase 2/3 clinical trials to test their efficacy in reducing COVID-19 mortality and morbidity are paxlovid (nirmatrelvir/ritonavir) and molnupiravir. Paxlovid is composed of a protease inhibitor and a pharmacokinetic enhancer and was tested in a phase 2/3 clinical trial. This study, performed on a cohort of 2246 patients, found that the treatment with nirmatrelvir and ritonavir reduced the risk of severe COVID-19 [33]. Another phase 3 clinical trial, performed on a cohort of 1433 patients, tested the efficacy of molnupiravir (a nucleoside analogue) and identified a reduced risk of hospitalization or death in early treated patients [34]. Other studies investigated the impact of IL-10 in SARS-CoV-2 infection. For example, a study performed in Italy on 85 PLWH found that they do not develop higher clinical severity or demonstrate increased risk for COVID-19. Moreover, HIV-1- and SARS-CoV-2-positive patients have higher levels of IL-10, thus suggesting an IL-10-mediated role in SARS-CoV-2 infection in PLWH [35]. Another important issue that has been poorly addressed is the prevalence of long-COVID-19 in PLWH taking cART. In a study conducted in India with a cohort of 94 PLWH, they found that the frequency of long-COVID-19 was 43.6% and that this is associated with moderate-severe SARS-CoV-2 infection [36]. Another study conducted in Italy on 123 PLWH with SARS-CoV-2 infection showed that the risk factors which can result in the development of post-acute COVID-19 syndrome are similar to those experienced by the general population such as severe COVID-19 and polypharmacy [37]. The discussed studies are summarized and reported in Table 2.

## 3. HIV-1 Therapy and SARS-CoV-2 Treatment

As mentioned in the previous section, the benefit of cART in PLWH who contract SARS-CoV-2 infection is still under investigation. A recent study compared a small number of PLWH infected with SARS-CoV-2 on cART with those not on cART. This paper found that COVID-19 infection had similar outcomes and inflammatory markers were high in both the studied groups [38]. In silico analyses indicate the potential of cART drugs binding SARS-CoV-2 protein targets [39].

Mahdi et al. tested the efficacy of various HIV-1 PIs, using a dark-to-bright fluorescent reporter gene for in vitro and cell-based assays. This strategy allows direct measurement of the activity of the analyzed protein; the reporter is quenched by a C-terminal region which is linked to the GFP via the protease substrate. The reporter gains fluorescence if the protease is active. All the drugs tested demonstrated inhibition efficacy in the micromolar range. HIV-1 PIs such as saquinavir, darunavir and atazanavir showed the best inhibitory activity, even though they all failed to completely inhibit the activity of the main protease of SARS-CoV-2 (M^pro^) in vitro [40]. Saquinavir also showed cytotoxic effect at high concentrations in comparison with the others.

A cytidine analogue, azvudine or FNC, is known for anti-retroviral activity and it has been recently approved for HIV-1 treatment in China [41,42]. FNC is active against viral proteins only upon phosphorylation (FNC-triphosphate). CL236 was used as an analogous of FNC-MP; it showed a reduction of coronavirus RNA production in vitro [42].

Some data suggested that drugs targeting HIV-1 reverse transcriptase (RT), or integrase (IN), could potentially inhibit SARS-CoV-2 entry. Using a receptor–anti-receptor binding assay (NanoLuc binary technology (NanoBiT), Lee et al. found that the RT inhibitor etravirine has an affinity for the receptor binding domain (RBD) of spike, blocking the binding of SARS-CoV-2 to the human ACE2 receptor [43]. A similar effect was observed with the IN inhibitor dolutegravir, but with a weaker efficacy compared with etravirine. However, these preliminary data were not confirmed in an in vitro infection system to test lentiviral-based pseudotyped virus neutralization due to inherent restrictions of lentiviral vector infection systems in the presence of IN and RT inhibitors [43]. Interestingly, treatment with another protease inhibitor, nelfinavir mesylate, showed considerable cell-to-cell fusion inhibition in vitro [44]. Indeed, it has been demonstrated that SARS-CoV-2 infection induces the formation of syncytia that may be driving the rapid spread of the virus in lung tissue [45]. These data on the efficacy of nelfinavir could potentially suggest new potential drug regimens to slow down the SARS-CoV-2 disease progression [44].

## 4. Novel Drugs to Resolve Co-Infections and Potentially to Guide the Development of Novel Anti-SARS-CoV-2 Therapies

Although limited information is currently known on SARS-CoV-2/HIV-1 co-infection due to the relative paucity of cases, this group of rare patients represents the opportunity to expand the discovery of currently used antiviral drugs and/or new ones with a broader spectrum of activity and to enhance the knowledge of the immunology of PLWH. Many anti-HIV-1 drugs were also repurposed for COVID-19 treatment as a first means to address the emergency.

Lopinavir and ritonavir (HIV-1 protease inhibitors), usually prescribed in combination, have been tested directly on patients since their positive effects against SARS-CoV and MERS-CoV infections. However, these studies have been abandoned since no clinical improvement was observed when compared with control groups [31,46,47]. In particular, ritonavir showed insufficient inhibitory activity [40]. Other HIV-1 PIs, nelfinavir and atazanavir, have also been proposed and tested in cellular models and showed better inhibition of the viral replication cycle [46]. However, data showed that nelfinavir requires a high dose to obtain inhibition of viral proteins. Atazanavir, on the other hand, is better tolerated by cells, although it also requires high concentrations [40]. Therefore, new drugs against SARS-CoV-2 in the presence or absence of HIV-1 infection are urgently required. The first step could be the identification of a possible cross-reactive virus target for drug development.

### 4.1. In Silico and In Vitro Identification of Viral Target to Resolve Co-Infections and Potentially to Guide the Development of Novel Anti-SARS-CoV-2 Drugs

Modern drug development usually uses in silico approaches to identify virus proteins that could possibly act as drug targets. In the case of SARS-CoV-2, potential targets are represented by the virus entry mechanism (spike-ACE2 interaction/fusion core blockade), the viral replication (RNA-dependent RNA polymerase, or RdRp) or the maturation processes (viral proteases) [48].

SARS-CoV-2, as with other coronaviruses, expresses a trimeric fusion protein on its envelope (spike protein, or S) [49,50]. The single monomer has separate subunits, namely S1 and S2. The N-terminal segment in the S1 region contains the RBD, which directly binds the ACE2 receptor on the host cells. The S2 region harbors the substrate sequence of proteases such as TMPRSS2, which promotes the fusion between the membranes [51,52]. The infectivity of the virus can be minimized or hindered by blocking its access to ACE2 and/or by preventing the formation of the fusion core by blocking the S1/S2 interface [43,53].

SARS-CoV-2 also requires RdRp to replicate its own genome into new copies for progeny virions [54]. SARS-CoV-2 RdRp is constituted by three domains. The drugs that interfere with the replication of the genome are designed to dock into the nucleotide binding cleft and to prevent RNA production, limiting the formation of replication competent virus particles [55].

The SARS-CoV-2 genome also encodes a long polyprotein that is subsequently processed into single functional proteins. Among these, the proteases PL^pro^ and 3CL^pro^ (or M^pro^) are fundamental in each of the maturation steps of the viral life cycle. PL^pro^ is also involved in deubiquitination to preserve viral proteins. M^pro^ has a pivotal role in polyprotein processing since viral maturation depends on M^pro^ activity [40,56].

Besides the identification of new potential drug targets, in the case of SARS-CoV-2, HIV-1 cART represented a starting point for virtual molecular analyses, molecular docking and interaction energies to predict interactions between viral proteins and available drugs to shorten the time required for the discovery of new therapies [57]. A small group of anti-HIV-1 drugs showed a cross-reaction to SARS-CoV-2, with clinically relevant results.

The envelope complex of HIV-1, gp160, contains a structure characterized by the presence of two heptad repeats (HRs), HR 1 in gp120 and HR2 in gp41. Once the envelope binds to the receptor, the subsequent conformational change induces the pairing of the HRs, promoting the fusion between membranes. From a structural point of view, the S1 and S2 subunits of the spike protein display a similar function [53,58]. Therefore, the effects of anti-HIV-1 drugs that abrogate the membrane fusion have been investigated. As an example, enfuvirtide (Enf) is one of the most common anti-fusion drugs administered for HIV-1 treatment. Since the emergence of SARS-CoV, it has been proposed as an anti-betacoronavirus drug due to evident similarities between SARS-CoV and HIV-1 envelopes. Enf showed high association levels with the structures of S2, since several residues that are relevant for enfuvirtide docking are conserved between gp41 and S2 [53]. A previous molecular study revealed the interaction between this drug and the spike protein of SARS-CoV-2, based on previous evidence from SARS-CoV. The study shows that the molecule impairs the transition from the pre-fusion to the fusion stage by docking in the interface between monomers [59].

The inhibitory potential of HIV-1 protease blockers against SARS-CoV-2 protease (M^pro^) was also investigated. MD studies revealed that TMB607 and TMC310911 (in combination with ritonavir, a drug usually included in cART therapy) could be candidates for SARS-CoV-2 M^pro^ blockade for future therapeutic applications [60,61]. Other anti-HIV-1 compounds were also tested for SARS-CoV-2 inhibition/interference based on molecular docking studies. Abacavir, fosamprenavir, indinavir and raltegravir resulted to better interact with SARS-CoV-2 fundamental proteins. Moreover, the group analyzed other predicted biological parameters, such as toxicity, sensitivity and mutagenicity, highlighting that the majority of these were non-mutagenic but potentially toxic [62]. Recently, a study by Y. Wu et al. investigated the binding energies and molecular docking properties of saquinavir, a direct HIV-1 protease inhibitor, on SARS-CoV-2 M^pro^. The group discovered that saquinavir is predicted as one of the best binders of M^pro^ [48].

### 4.2. Attempts to Resolve Co-Infection by Enhancing a Humoral Response: The Identification of Possible Broadly Neutralizing Antibodies against HIV-1 and SARS-CoV-2

Previous studies revealed shared motifs in the structure of HIV-1 and SARS-CoV-2 envelope viral proteins [49,58]. Recent studies have aimed to identify antibodies against SARS-CoV-2 in PLWH. Broadly neutralizing antibodies (bnAbs), which produce highly neutralizing responses, are under evaluation for cross-reactivity against SARS-CoV-2. In one study, by adopting an enzyme-linked immunoassay (ELISA) screening approach, six bnAbs were selected to test their neutralization ability in vitro through SARS-CoV-2 pseudotyped virus neutralization assay (PVNA). Only one bnAb showed a sufficient neutralization capacity against SARS-CoV-2, but failed to block infection in a live-virus assay [63]. In another study, a cohort of SARS-CoV-2-recovered patients were enrolled and screened to identify the presence of nAbs through PVNA. In this case, the isolated nAbs were also tested in vivo in a Syrian hamster animal model. The study concluded that only a portion of the tested nAbs confer protection against the SARS-CoV-2 disease [64].

A recent study reported a similar finding. In this case, the group found cross-reactivity against an HIV-1 envelope in sera from SARS-CoV-2-spike immunized mice via ELISA. The opposite situation (anti-HIV-1 Env antibodies against SARS-CoV-2 spike) was also confirmed by ELISA analyses. However, while anti-SARS-CoV-2 sera neutralized the live virus, they failed to induce strong infectivity reduction against the HIV-1-pseudotyped virus. Moreover, the anti-HIV-1 sera failed to block the entry of live SARS-CoV-2 in a cytopathic effect (CPE) assay [65].

A recent study investigated the cross-reactivity of monoclonal anti-HIV-1 antibodies against the epitopes of SARS-CoV-2 spike glycans [66]. Cross-reactivity against viral proteins that carry host’s glycans is rare but possible. Part of the tested antibodies resulted cross-reactively in ELISA tests and Western-blot analysis (both native and denatured substrates) but failed at neutralizing the SARS-CoV-2-pseudotyped virus. The group concluded that the cross-reactivity was mediated by glycan moieties, since the use of glycan-rich casein-based buffers abrogated the cross-reaction [66].

## 5. Conclusions

Following two years of the outbreak of SARS-CoV-2, there is a solid and wide body of the literature that explores the risk of SARS-CoV-2 infection and/or COVID-19 disease progression in the general population. On the other hand, studies on the risk of infection and severe disease in the presence of persistent co-infections such as HIV-1 are less abundant and therefore deserve particular attention [67,68]. In parallel, the development of in vitro studies to provide robust methodological approaches to study co-infections is still ongoing. In this review, we have provided a global overview on the implications and clinical management of SARS-CoV-2 and HIV-1 co-infection, as well as in the field of novel anti-retroviral drug development. This study of the literature seems to suggest that HIV-1 infection is not an independent factor of a higher risk of SARS-CoV-2 infection or a worse clinical outcome compared with the HIV-1-negative population. Whilst the putative protective role of cART against a more severe COVID-19 clinical outcome still needs to be confirmed, it seems clear that the past 20 years of research in the HIV-1 field have set a solid ground for drug development studies.

## Figures and Tables

**Table 2:** Summary of relevant in vivo studies studying HIV-1 and SARS-CoV-2 co-infections.

First Author and Year	Country	Studied Cohorts	Results
Isernia et al., 2020 [24]	France	30 HIV-1 and SARS-CoV-2 positive patients	HIV is not an independent risk factor for SARS-CoV-2.
Noe et al., 2021 [25]	Germany	500 PLWH	HIV-1 is not associated with elevated probability of SARS-CoV-2 infection
Härter et al., 2020 [26]	Germany	33 HIV-1 and SARS-CoV-2 positive patients	PLWH on cART regimen did not show increased risk of mortality and morbidities when experiencing symptomatic COVID-19 infection
Vizcarra et al., 2020 [27]	Spain	51 HIV-1 and SARS-CoV-2 positive patients	HIV-1 infection is not associated with protection and lower risk of severe SARS-CoV-2 infection
Geretti et al., 2021 [28]	United Kingdom	47,592 SARS-CoV-2 patients (0.26% PLWH)	There is an association between PLWH under 60 years old and an increased SARS-CoV-2 mortality compared with the HIV-1 uninfected group
Vanetti et al., 2021 [35]	Italy	85 PLWH among whom 4 had SARS-CoV-2 infection	HIV-1 is not associated with increased risk and severity of SARS-CoV-2 infection. Moreover, higher levels of IL-10 in HIV-1 and SARS-CoV-2 positive patients were observed, thus suggesting an IL-10 role in SARS-CoV-2 infection in PLWH
Del Amo et al., 2020 [29]	Spain	77,590 PLWH under cART regimen among whom 236 with SARS-CoV-2 infection	HIV-1 positive man older than 70 years old possess a higher risk for COVID-19 diagnosis. TDF/FTC cART regimen seems to be protective for COVID-19 and COVID-19-related hospitalization
Cao et al., 2020 [31]	China	199 SARS-CoV-2 patients to test efficacy of PIs	There is no improvement in SARS-CoV-2 infection in patients taking standard drug regimen supplemented with ritonavir/lopinavir compared with patients taking the standard drug regimen. Ritonavir/lopinavir treatment had no antiviral effect on SARS-CoV-2-infected patients
Ye et al., 2020 [32]	China	47 SARS-CoV-2 patients to test efficacy of PIs	The protease inhibitors ritonavir/lopinavir in combination with pneumonia adjuvant drugs have beneficial effects in the management of COVID-19 symptoms.

## Data Availability

Not applicable.
